# Key features of neural variability emerge from self-organized sequence learning in a deterministic neural network

**DOI:** 10.1186/1471-2202-16-S1-P266

**Published:** 2015-12-18

**Authors:** Christoph Hartmann, Andreea Lazar, Jochen Triesch

**Affiliations:** 1 Frankfurt Institute for Advanced Studies (FIAS), Frankfurt, Germany; 2 Ernst-Strüngmann Institute (ESI), Frankfurt, Germany

## 

Cortical responses to identical stimuli show high trial-to-trial variability. This variability is commonly interpreted as resulting from internal noise. However, much of the variability can be explained by the pre-stimulus spontaneous activity [1]. In fact, the contribution of this spontaneous activity to the evoked response is sufficiently strong to bias perceptual decisions [2]. Importantly, spontaneous activity is structurally similar to evoked activity [3] and this similarity may be the result of learning an internal model of the environment during development [4]. Consistent with this idea, spontaneous activity seems to be a superset of possible evoked responses [5] and trial-to-trial variability drops at stimulus onset [6]. At present, it is unclear how these features of neural variability arise in cortical circuits.

Here, we show that all of these phenomena emerge in a completely deterministic self-organizing recurrent network (SORN) model [7]. The network consists of recurrently connected excitatory and inhibitory populations of McCulloch-Pitts units. The dynamics are shaped by spike-timing dependent plasticity (STDP) and homeostatic plasticity mechanisms in response to structured input sequences. After a period of self-organization, during which the network learns an internal model of the input sequences, we observe all phenomena mentioned above: evoked responses and perceptual decisions can be predicted from prior spontaneous activity, spontaneous activity outlines the realm of evoked responses, Fano factors drop at stimulus onset, and spontaneous activity closely matches evoked activity patterns. In addition, the network produces the common signs of Poissonian variability in single units.

In sum, our model demonstrates that key features of neural variability emerge in a fully deterministic network from self-organized sequence learning via the interaction of STDP and homeostatic plasticity mechanisms. These results suggest that the high trial-to-trial variability of neural responses need not be taken as evidence for noisy neural processing elements.

**Figure 1:**
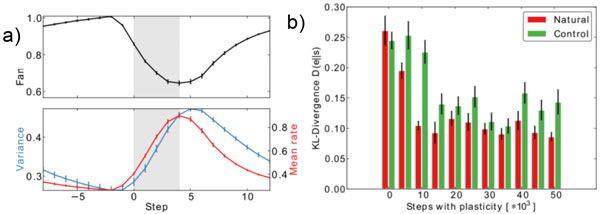
**Two example results after self-organization**. a) The neural variability drops at stimulus onset. b) Spontaneous and evoked activity become more similar during learning

## References

[B1] Arieli A Sterkin A Grinvald A Aertsen A Dynamics of ongoing activity: explanation of the large variability in evoked cortical responses Science 1996 273 1868 1871 8791593 10.1126/science.273.5283.1868

[B2] Hesselmann G Kell C a Eger E Kleinschmidt A Spontaneous local variations in ongoing neural activity bias perceptual decisions Proc Natl Acad Sci U S A 2008 105 10984 10989 18664576 10.1073/pnas.0712043105 PMC2504783

[B3] Kenet T Bibitchkov D Tsodyks M Grinvald A Arieli A Spontaneously emerging cortical representations of visual attributes Nature 2003 425 954 956 14586468 10.1038/nature02078

[B4] Berkes P Orbán G Lengyel M Fiser J Spontaneous cortical activity reveals hallmarks of an optimal internal model of the environment Science (80- ) 2011 331 83 87 10.1126/science.1195870 PMC3065813 21212356

[B5] Luczak A Barthó P Harris KD Spontaneous events outline the realm of possible sensory responses in neocortical populations Neuron 2009 62 413 425 19447096 10.1016/j.neuron.2009.03.014 PMC2696272

[B6] Churchland MM Stimulus onset quenches neural variability: a widespread cortical phenomenon Nat Neurosci 2010 13 369 378 20173745 10.1038/nn.2501 PMC2828350

[B7] Lazar A Pipa G Triesch J Emerging Bayesian priors in a self-organizing recurrent network Artificial Neural Networks and Machine Learning - ICANN 2011 127 134

